# Effects of Fertility on Gene Expression and Function of the Bovine Endometrium

**DOI:** 10.1371/journal.pone.0069444

**Published:** 2013-08-05

**Authors:** Megan A. Minten, Todd R. Bilby, Ralph G. S. Bruno, Carolyn C. Allen, Crystal A. Madsen, Zeping Wang, Jason E. Sawyer, Ahmed Tibary, Holly L. Neibergs, Thomas W. Geary, Stefan Bauersachs, Thomas E. Spencer

**Affiliations:** 1 Department of Animal Sciences, Washington State University, Pullman, Washington, United States of America; 2 Texas AgriLife Research and Extension, Stephenville, Texas, United States of America; 3 West Texas A&M University and Texas AgriLife Research and Extension, Amarillo, Texas, United States of America; 4 USDA-ARS, Fort-Keogh, Miles City, Montana, United States of America; 5 Department of Animal Science, Texas A&M University, College Station, Texas, United States of America; 6 Department of Veterinary Clinical Sciences, College of Veterinary Medicine and Center for Reproductive Biology, Washington State University, Pullman, Washington, United States of America; 7 Laboratory for Functional Genome Analysis (LAFUGA), Gene Center, Ludwig-Maximilians-Universität München, Munich, Germany; The University of Georgia, United States of America

## Abstract

Infertility and subfertility are important and pervasive reproductive problems in both domestic animals and humans. The majority of embryonic loss occurs during the first three weeks of pregnancy in cattle and women due, in part, to inadequate endometrial receptivity for support of embryo implantation. To identify heifers of contrasting fertility, serial rounds of artificial insemination (AI) were conducted in 201 synchronized crossbred beef heifers. The heifers were then fertility classified based on number of pregnancies detected on day 35 in four AI opportunities. Heifers, classified as having high fertility, subfertility or infertility, were selected for further study. The fertility-classified heifers were superovulated and flushed, and the recovered embryos were graded and then transferred to synchronized recipients. Quantity of embryos recovered per flush, embryo quality, and subsequent recipient pregnancy rates did not differ by fertility classification. Two in vivo-produced bovine embryos (stage 4 or 5, grade 1 or 2) were then transferred into each heifer on day 7 post-estrus. Pregnancy rates were greater in high fertility than lower fertility heifers when heifers were used as embryo recipients. The reproductive tracts of the classified heifers were obtained on day 14 of the estrous cycle. No obvious morphological differences in reproductive tract structures and histology of the uterus were observed in the heifers. Microarray analysis revealed differences in the endometrial transcriptome based on fertility classification. A genome-wide association study, based on SNP genotyping, detected 7 moderate associations with fertility across 6 different chromosomes. Collectively, these studies support the idea that innate differences in uterine function underlie fertility and early pregnancy loss in ruminants. Cattle with defined early pregnancy success or loss is useful to elucidate the complex biological and genetic mechanisms governing endometrial receptivity and uterine competency for pregnancy.

## Introduction

Infertility and subfertility are important and pervasive problems in both domestic animals and humans, and the greatest limitation to reproductive efficiency across mammalian species is embryonic mortality [1]. Embryo survival is a major factor affecting production and economic efficiency in all systems of meat and milk production by ruminants [2]–[4]. In beef cattle, estimates indicate that fertilization rate for oocytes is 90%, whereas average calving rates to a single service are between 40% and 55%, suggesting a rate of embryonic/fetal mortality (excluding fertilization failure) of about 35% to 50% [5]. The majority of embryonic loss (70–80%) occurs in the first 3 weeks of pregnancy [6], particularly between days 7 and 16 of pregnancy [7]–[9]. Further, embryo mortality is greater in non-lactating cows than heifers [7], and early pregnancy loss is even greater in high producing lactating dairy cattle and can approach 70% [2], [10], [11]. Infertility and subfertility are also major cost factors in the cattle embryo transfer industry [12]. Mean survival rate to calving following transfer of *in vivo*-derived embryos from superovulated donors is only 43% with a range from 31% to 60% [13], whereas the mean survival rate after transfer of *in vitro*-produced (IVP) embryos is lower and ranges from 30% to 40% [4], [13]. Pregnancy loss is the most common complication of human gestation, occurring in as many as 75% of all women trying to have children. Roughly one-half of conceptions in humans result in pregnancy loss, with losses occurring most frequently in the first two weeks of gestation [14]. The failure to establish pregnancy in humans and animals is due to both embryonic and maternal factors [13], [15], [16]. Many of the pregnancy losses observed in natural or assisted reproductive technology pregnancies can be attributed to inadequate endometrial receptivity, which can be defined as the physiological state of the uterus when conceptus growth and implantation for establishment of pregnancy is possible. However, knowledge of the complex biological and genetic mechanisms governing endometrial receptivity and conceptus implantation is limited in both domestic ruminants and humans [17].

After conception (day 0) in cattle, the embryo enters the uterus at the morula stage on days 4 to 5 of gestation and develops into a blastocyst surrounded by a zona pellucida. After hatching from the zona pellucida on days 9 to 10, the spherical blastocyst (∼0.5 mm) begins to grow and changes from a spherical to ovoid shape between days 12 and 14 during a transitory phase preceding elongation and is now termed a conceptus (embryo and associated extra-embryonic membranes) [18]. Ovoid conceptuses are about 2 mm on day 13, reach a length of about 60 mm (6 cm) by day 16, and are 20 cm or more by day 19. Indeed, the blastocyst/conceptus doubles in length every day between days 9 and 16 with a significant increase (∼10–fold) in growth between days 13 and 14 [7]. After day 16, the elongating conceptus begins the process of implantation and placentation [19]. Blastocyst survival and growth and elongation of the conceptus does not occur *in vitro* as it is dependent on ovarian progesterone and secretions supplied by the endometrium of the uterus [18]. Progesterone, from the ovarian corpus luteum, acts on the endometrium to regulate conceptus growth and elongation [20], which is critical for production of interferon tau (IFNT) [21], [22]. Interferon tau is the conceptus derived signal for maternal recognition of pregnancy that acts on the endometrium to inhibit production of luteolytic prostaglandin F2α (PGF), thereby sustaining continued production of progesterone by the corpus luteum of the ovary [23]. Inadequate development of the conceptus results in low IFNT production, inability to maintain the corpus luteum, and early pregnancy loss [24]. Although much information is known about embryo development into a blastocyst from *in vitro* systems [25], very little is known about post-hatching blastocyst growth and conceptus development in cattle [26]. Available evidence supports an unequivocal role for endometrial secretions of the uterus as primary regulators of conceptus survival, growth and development throughout pregnancy (reviewed in [26]–[28]). Endometrial epithelial secretions are particularly important for conceptus survival and growth, as uterine gland knockout (UGKO) ewes display recurrent early pregnancy loss due to a defect in conceptus elongation that manifests between days 12 and 14 of pregnancy [22], [29]. Uterine secretions in the lumen are a complex mixture of proteins, amino acids, sugars, lipids and ions that are derived from genes expressed in the endometrium as well as selective transport of components (amino acids, glucose, albumin and other proteins) from maternal blood [30], [31]. Proteins in histotroph of the bovine uterus are not well defined, but include enzymes, growth factors, cytokines, adhesion proteins, and transport proteins [20], [26]. Although much is known about gene expression changes during the estrous cycle and early pregnancy of cattle, the essential endometrial genes and secretions that mediate post-hatching blastocyst growth and conceptus elongation have not been determined [20], [26], [32], [33].

One of the major impediments to research on the genetics and physiology of early pregnancy in cattle is the lack of animals with defined high and low rates of early pregnancy loss. Improvement of functional traits using conventional approaches of quantitative genetics is difficult, because most reproductive traits are complex (polygenic) with low heritability [34], [35]. McMillan and Donnison [36] summarized a novel approach for experimentally identifying high and low fertility heifers based on early pregnancy success. The approach was to use serial ET of *in vitro*-produced (IVP) embryos followed by pregnancy determination on day 35 and then termination. Out of 200 heifers, the investigators identified 25 heifers with high (76%) and low (11%) aggregate pregnancy rates. Of particular relevance, they suggested that a failure in the mechanism involved in conceptus elongation and maternal recognition of pregnancy was a major cause of early pregnancy loss in low fertility heifers [36], [37]. Accordingly, the selected high fertility heifers would have a uterus that was superior in the ability to support growth and development of the conceptus. The present study tested the hypothesis that a similar experimental approach to exploit natural variation in early pregnancy rates in beef heifers can be used to study early pregnancy loss and success. The approach was to identify subpopulations of cattle with contrasting early pregnancy rates using an approach similar to that originally described by McMillan and Donnison [36], except that 4 serial rounds of artificial insemination (AI) were used to classify heifers as high or low fertility based on pregnancy outcomes. The fertility-classified heifers were used in a series of experiments to begin deciphering the biological and genetic mechanisms governing endometrial receptivity and pregnancy loss.

## Materials and Methods

### Animal Handling, Artificial Insemination and Embryo Transfer

All animal procedures were conducted in accordance with the Guide for the Care and Use of Agriculture Animals in Research and Teaching and approved by the Institutional Animal Care and Use Committees of Texas A&M University, USDA-ARS Fort Keogh Livestock and Range Research Laboratory, and Washington State University.

#### Artificial Insemination

**Figure 1:**
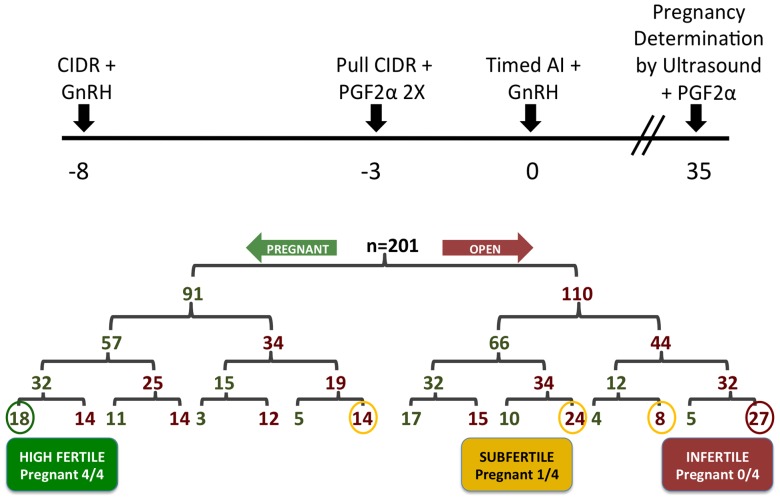
Experimental design and results for selection of beef heifers for uterine capacity for early pregnancy using a serial timed artificial insemination (AI) approach. See text for detailed description of results.

Crossbred pubertal beef heifers (approximately ¼ *Bos indicus* and ¾ *Bos taurus*) of 14–15 months of age (n = 201) from the Texas AgriLife McGregor Beef Cattle Research Center were synchronized for AI using the 5-day Co-Synch program [38]. Heifers, observed in standing estrus at 48 hours post-CIDR (controlled intravaginal drug releasing device) removal, were bred by AI using semen from a single sire at 60 hours post-CIDR removal and a single technician. All remaining heifers were bred by AI using semen from the same single sire at 72 hours post-CIDR removal by one of three rotating technicians. Pregnancy determinations were made on days 35 to 38 of pregnancy using transrectal ultrasonography for detection of the conceptus. All heifers were given 25 mg PGF2α (Lutalyse; Pfizer, Kalamazoo, MI) to terminate pregnancy and/or synchronize estrus following ultrasonography. This synchronization and AI protocol was repeated an additional three times, providing all heifers four opportunities to conceive (Fig. 1).

#### Embryo Transfer Using Fertility-classified Heifers as Donors

A subset of the fertility-classified heifers (HF, n = 14; SF, n = 14; IF, n = 11) was used to differentiate between uterine and oocyte causes of infertility. All heifers received 25 mg PGF2α (i.m.) on day -17 followed by 100 μg GnRH (Factrel, i.m.; Pfizer Animal Health) on day -14. On day -8, heifers received a CIDR and an i.m. injection of 2.5 mg estradiol-17 β benzoate and 50 mg progesterone. From days -4 to -1, all heifers received follicle-stimulating hormone (Folltropin-V, Bioniche Animal Heath, Athens, GA) twice daily i.m. in the following doses: 50 mg on day -4; 50 mg on day -3; 28 mg on day -2; and 14 mg on day -1. On day -2 PM and day -1 AM, heifers were given an i.m. injection of 25 mg PGF2α. The CIDR was removed on day -1 AM. All heifers were bred by AI using semen from the same sire on day 0 PM and day 1 AM. Those heifers not exhibiting estrus were given 100 μg GnRH on day 1 AM at AI. Nonsurgical embryo collection was performed on day 7 by a single technician in heifers (n = 32) that a flush catheter could be traversed through the cervix. Embryos were evaluated (quantity, developmental stage, and quality) by a single technician using the standards set forth by the International Embryo Transfer Society (Savoy, IL). The best embryos were selected and fresh transfer performed in synchronized recipient cows.

Crossbred multiparous beef cows (n = 108) from the USDA-ARS Fort Keogh herd were synchronized for embryo recipients using a similar protocol as heifers had received with the exception that recipients did not receive FSH administration. In addition, recipient cows received twice the dose of estradiol benzoate and progesterone as heifers on day -8 and PGF2α and CIDR removal on day -2. If estrus was not detected within 72 hours following CIDR removal and PGF2α, cows received 100 μg GnRH to induce ovulation. A single superovulated embryo from the fertility classified donor heifers was transferred nonsurgically into the uterine horn ipsilateral to the CL on day 7 in recipient cows. Pregnancy was diagnosed on day 36 of gestation using transrectal ultrasonography and confirmation of an embryo heartbeat.

#### Embryo Transfer Using Fertility-classified Heifers as Recipients

Fertility-classified heifers (HF, n = 14; SF, n = 14; IF, n = 11) whose estrous cycles were presynchronized, received 25 mg PGF2α i.m. on day 16 of the estrous cycle. Heifers were observed for estrus and two frozen-thawed, in vivo produced morula or early blastocyst embryos with a quality grade of excellent/good or moderate were nonsurgically transferred into the ipsilateral horn to the CL on day 7 after estrus. Pregnancy determinations were made on day 25–27 after transfer (days 32 to 34 of gestation) using transrectal ultrasonography for detection of embryo(s) heartbeat.

### Blood Collection and Progesterone Radioimmunoassay

Blood samples were collected from the fertility-classified heifers (n = 39) to characterize serum concentrations of progesterone during the estrous cycle after estrous synchronization. Heifers received 100 μg GnRH im and a CIDR on day -9 and CIDR removal and 25 mg PGF2α i.m. on day -3 to synchronize estrus. Using only those heifers (n = 35) that exhibited estrus, blood was collected by coccygeal or jugular venipuncture into 10 ml tubes (BD Vacutainer, Franklin Lakes, NJ) during the estrous cycle on days 3, 5, 7, 8, 9, 10, 11, 12, 14 and 16 of the estrous cycle. Blood samples were placed at 4°C for 24 hours, followed by centrifugation at 1,200×g for 25 minutes at 4°C. Serum was collected and stored at −20°C until progesterone radioimmunoassay (RIA) was performed. Progesterone was analyzed in all serum samples by RIA (Coat-a-Count tubes, Diagnostic Products Corporation, Los Angeles) with a previously validated assay [39]. The inter- and intra-assay CV were 1.82% and 1.5%, respectively and assay sensitivity was 0.08 ng/mL.

### Tissue Collection

Estrus was synchronized in the subset of fertility-classified heifers as described above, and heifers were harvested on day 14 of the estrous cycle (day 0 = estrus). At harvest, the uterine lumen was flushed with 20 ml of 10 mM Tris (pH 7.2). The volume of uterine flush was measured and recorded, then clarified by centrifugation (3,000×g at 4°C for 15 min). Supernatant was carefully removed with a pipet, aliquoted and placed on dry ice until storage at −80°C was permitted. Uterine sections (∼0.5 cm) were fixed in fresh 4% paraformaldehyde in PBS (pH 7.2) and also embedded in OCT compound (Tissue-Tek, Torrance, CA). The remaining endometrium (both caruncular and intercaruncular) was physically dissected from the myometrium and minced with a pair of scissors. Endometrium, myometrium, and corpus luteum tissue was placed on dry ice until permanent storage at −80°C for subsequent RNA extraction.

### Microarray Analysis

Total cellular RNA was isolated from frozen endometrium using Isol-RNA lysis reagent (5 Prime, Gaithersburg, MD) according to manufacturer's instructions and stored at −80°C in nuclease-free water. RNA concentration was determined by spectrophotometry (NanoDrop Technologies, Wilmington, DE). Isolated endometrial total RNA was treated with DNase I (Qiagen, Valencia, CA) and processed with RNeasy Mini Kit (Qiagen) according to manufacturer's instructions to remove genomic DNA. RNA quality was then assessed using the Agilent Bioanalyzer 2100 (Agilent Technologies, Santa Clara, CA) and RNA 6000 Nano Labchip kit (Agilent Technologies) according to manufacturer's instructions. Microarray hybridization was conducted on the EmbryoGENE bovine microarray [40]. Preparation of cDNA was performed according to Agilent's one-color microarray-based gene expression analysis instructions. Briefly, 150 ng total RNA from each heifer was amplified by T7 RNA polymerase and labeled with cyanine 3-labeled CTP (Agilent Technologies). Antisense cRNA (1650 ng) was hybridized on the Agilent-manufactured EmbryoGENE slides. Microarray slides (n = 8) were hybridized for 17 hours at 65°C, then washed for 1 min in gene Expression Wash Buffer 1 at room temperature, 3 min in gene Expression Wash Buffer 2 at 42°C, 10 sec in 100% acetonitrile at room temperature, and 30 sec in Stabilization and Drying Solution (Agilent Technologies). Slides were scanned with the PowerScanner (Tecan, San Jose, CA), and data extraction was performed with Array-Pro Analyzer 6.3 (MediaCybernatics, Bethesda, MD). The data discussed in this publication have been deposited in NCBIs Gene Expression Omnibus (GEO, http://www.ncbi.nlm.nih.gov/geo/) and is accessible through GEO Series accession number GSE46274.

Probes were filtered based on background-corrected signals to remove probes that without hybridization signals above background levels. Probes which passed the filter needed to have background-corrected signals of >9 in the samples of at least one of the experimental groups (in at least 11 samples out of 14 in the ‘HF’ group, in at least 7 samples out of 8 in the ‘IF’ group, or in at least 11 samples out of 14 in the ‘SF’ group, respectively). Signal intensities of these probes were subsequently normalized with the BioConductor package ‘VSN’ [41]. A heatmap based on pair-wise distances (BioConducter package ‘Geneplotter’) was generated for quality control of the samples. Significance analysis was performed using the BioConductor package ‘Limma’. Hierarchical clustering was performed by the use of the HCL function of MeV software (v.4.8.1, TM4 software suite). Integrated analysis of different functional databases was done using the “Functional annotation clustering” tool of the Database for Annotation, Visualization, and Integrated Discovery (DAVID) [42].

### Reverse Transcription and Quantitative Real-time PCR

Total RNA from each DNase treated sample was reverse transcribed. Briefly, total RNA (2 μg) was combined with oligo(dT) primer (0.5 μg/ml, Promega, Madison, WI), mixture of deoxynucleotides (10 mM each, Promega) and incubated at 65°C for 5 min. A reverse transcription mixture containing 5X first-strand buffer, 0.1 M dithiothreitol and SuperScript II reverse transcriptase (Invitrogen, Carlsbad, CA) was added to the reaction to yield a 20 μl volume. Reverse transcription was performed under the following conditions: 25°C for 10 min; 42°C for 60 min; and 70°C for 5 min. Genomic DNA contamination was tested by inclusion of mixtures without reverse transcriptase. Resulting cDNA was stored at −20°C for further analysis.

Real-time analysis was performed using the ABI 7500 system (Applied Biosystems, Carlsbad, CA) with Power SYBR Green PCR Master Mix (Applied Biosystems) or the Bio-Rad CFX96 with SSOAdvanced SYBR Green (Bio-Rad, Hercules, CA). Specific oligonucleotide primers were designed and analyzed with Oligo 7 (Molecular Biology Insights, Cascade, CO), and sequences are summarized in Supplementary Table S3. Primer specificity and efficiency was evaluated using an amplification run with dissociation curve, ensuring that a single product was amplified and efficiency met (−3.6> slope > −3.1). PCR without template was used as a negative control to verify experimental results. The threshold line was set in the linear region of the amplification plots above the baseline noise, and threshold cycle (CT) values were determined as the cycle number in which the threshold line intersected the amplification curve. The bovine *RPL19* gene was used as a reference gene and ran on both real-time PCR platforms.

### Genome-wide Association Study (GWAS)

DNA was extracted from blood using the Qiagen DNA extraction kit, and genotyped using the Illumina BovineHD BeadChip by GeneSeek (Lincoln, NE). The GWAS using the SNP genotyping data was conducted with PLINK [43] using methods described previously [44]. Multiple testing corrections were based on modified Wellcome Trust recommendations that accounted for array size.

### Statistical Analyses

All statistical analyses were performed using Statistical Analysis Software (SAS Institute Inc., Cary, NC). Total quantity of embryos recovered in flush of donor heifer and quantity of transferrable embryos (deemed as stage ≥4 morula, with quality grade <3) in flush were subjected to least-squares analyses of variance (ANOVA) using the PROC GLM procedure in SAS, with donor phenotype (high fertile, subfertile or infertile) as the fixed variable. Pregnancy outcome of the recipient cows receiving an embryo from donor heifers was analyzed by Chi Square analysis using the PROC GENMOD procedure in SAS, with donor phenotype as the fixed variable. Changes in serum concentrations of progesterone over the estrous cycle were analyzed by ANOVA for repeated measures using the PROC MIXED procedure in SAS. Pregnancy outcome of heifers receiving embryos was analyzed by Chi Square analysis using the PROC GENMOD procedure in SAS, with heifer phenotype as the fixed variable. Heifer serum concentration of progesterone at embryo transfer was analyzed by ANOVA using the PROC GLM procedure in SAS, with heifer phenotype as the fixed variable. For the analysis of quantitative data from real-time PCR, CT values were subjected to ANOVA using the PROC GLM procedure to analyze effect of phenotype with *RPL19* values used as a covariate. All tests of significance were performed using the appropriate error terms according to the expectation of the mean squares for error. Significance was considered to be *P*≤0.05. Data are presented as the least-squares mean (LSM) and SEM.

## Results

### Classification of Heifers for Fertility Using Artificial Insemination

Pubertal crossbred beef heifers (n = 201) were given four opportunities to establish pregnancy using ovulation synchronization and AI followed by pregnancy determination on day 35 (Fig. 1). Heifers were then classified according to aggregate pregnancy outcome as being high fertile (HF, pregnant 4 of 4 opportunities; n = 18), subfertile (SF, pregnant 1 of 4 opportunities; n = 46), or infertile (IF, pregnant 0 of 4 opportunities; n = 27).

### Pregnancy Rates of Fertility-classified Heifers as Embryo Donors and Recipients

In order to evaluate oocyte fertility and function related to pregnancy, a subset of the fertility-classified heifers (HF, n = 14; SF, n = 14; IF, n = 11) were superovulated and flushed to recover embryos. The best 3 recovered embryos from each heifer (when more than one embryo was recovered) of high quality (stage 4 or 5, grade 1 or 2) were then transferred into synchronized recipient cows (n = 73). As summarized in Table 1, the number of collected embryos and transferrable embryos was not different (*P*>0.10) among the fertility-classified heifers. The pregnancy per ET of the recipient cows averaged 51% and was not associated (*P*>0.10) with fertility classification of the donor heifers.

**Table 1:** Summary of an embryo transfer experiment using fertility classified heifers as embryo donors.

Fertility Class	Number	Embryos/Oocytes Recovered^a^	Range	Transferrable Embryos^a^	Range	Total Embryos Transferred	Pregnancy Rate of Recipient Cows
High Fertile (HF)	13	13.5+2.7	1–40	7.5+1.8	0–20	28	54%
Subfertile (SF)	13	13.6+2.7	2–31	5.6+1.8	0–10	32	39%
Infertile (IF)	6	9.5+3.9	1–30	4.6+2.7	0–22	13	62%

a Mean ± SEM.

In order to evaluate uterine capacity for pregnancy, two high quality *in vivo* produced embryos (stage 4 or 5, grade 1 or 2) were transferred into fertility-classified heifers (HF, n = 13; SF, n = 13; IF, n = 11). Two heifers (one HF and one SF) were not detected in estrus and thus did not receive embryos. Pregnancy rate per ET, determined on days 32 to 34 of gestation by ultrasound, was greater (*P* = 0.04) for the HF heifers (69%) than IF heifers (27%) and tended (*P* = 0.10) to be greater for the HF (69%) than SF heifers (39%). Pregnancy rate per ET was not different (*P* = 0.56) between the SF and IF heifers.

### Post-ovulatory Serum Progesterone Profile, Histology of the Uterus, and Morphology of the Reproductive Tract is Not Different in Fertility-classified Heifers

**Figure 2:**
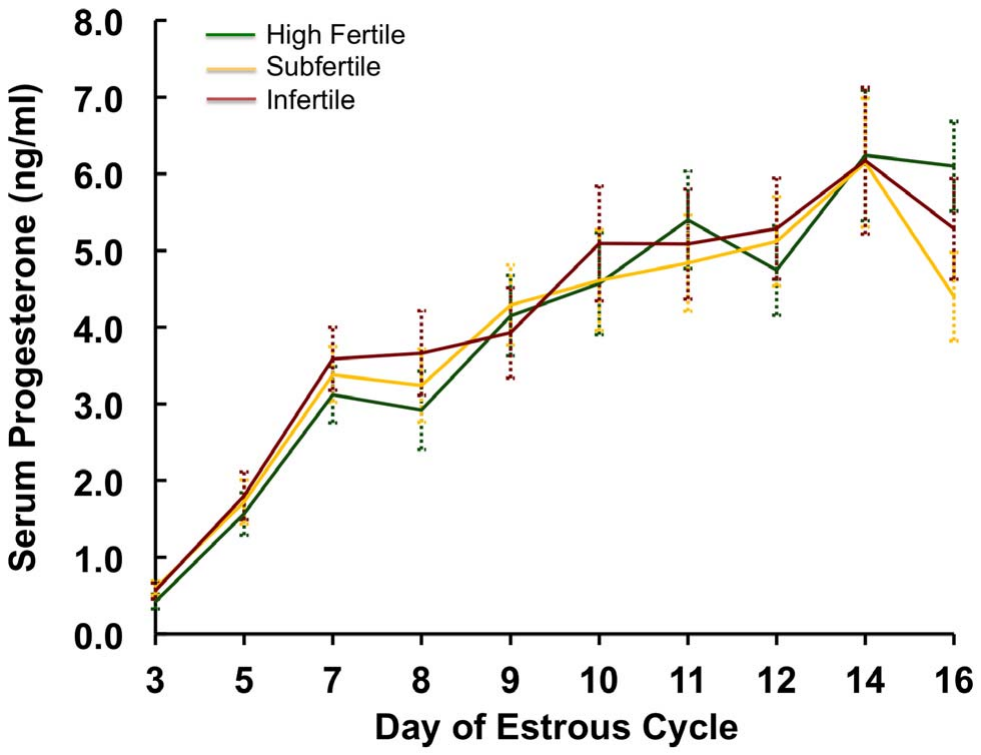
Circulating concentrations of progesterone in a subset of fertility classified heifers after ovulation. Note the lack of difference in serum progesterone levels in high fertile (HF, n = 13), subfertile (SF, n = 12) and infertile (IF, n = 10) heifers.

Progesterone influences elongation of the bovine conceptus during early pregnancy, and conceptus growth depends on the post-ovulatory rise in progesterone [45], [46]. As illustrated in Fig. 2, the post-ovulatory rise in circulating concentrations of progesterone was not different (*P*>0.10) between fertility-classified heifers after estrus. In order to obtain the reproductive tract, all heifers were synchronized to estrus, and the tract was harvested on day 14 post-estrus. Gross morphology of all reproductive tract tissues was not overtly different between the heifers (*data not shown*). All heifers had two uterine horns, oviducts and ovaries and a cervix. The histology of the uterus, assessed by hematoxylin- and eosin-stained cross-sections of the uterine horns, was also not different (*data not shown*); all uteri contained an endometrium with histologically normal cell types in appropriate numbers, e.g. all contained endometrial glands without evidence of infection.

### Endometrial Gene Expression is not Substantially Different in Fertility-classified Heifers

**Figure 3:**
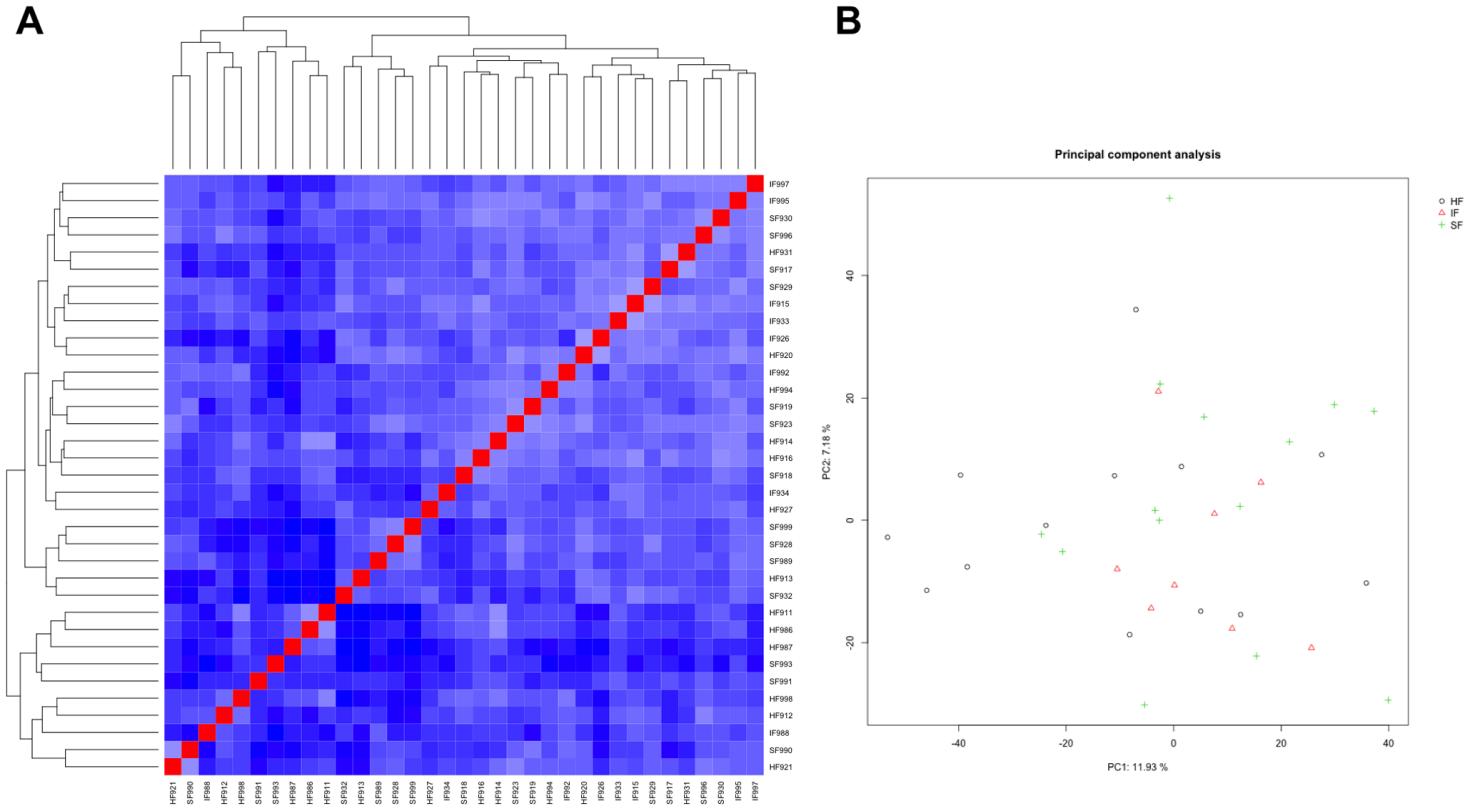
Heatmap of pairwise correlations and principal component analysis (PCA) of microarray data. (A) Microarray data were filtered for detectable probes and normalized with the BioConductor package vsn. Normalized data were used for calculation of pairwise distances and drawing of a heatmap by use of the BioConductor package geneplotter. Each column represents one sample and shows the correlation to all samples (including itself), with red for correlation  = 1 and blue for the lowest observed correlation. Note the clear homogeneity in the samples from fertility classified heifers (HF, high fertile; SF, subfertile; IF, infertile). (B) PCA is a plot distribution indicating the source of greatest variation in the overall transcriptional profiles of the samples. Each symbol represents one replicate. Note the clear lack of separation of samples based on fertility classifications (HF, high fertile; SF, subfertile; IF, infertile).

In order to ascertain potential functional differences in the endometria of fertility-classified heifers, transcriptional profiling of the endometria from fertility-classified heifers (HF, n = 14; SF, n = 14; IF, n = 11) was conducted using the EmbryoGENE bovine microarray [40], which is comprised of 42,242 total probes including 21,139 known reference genes, 9,322 probes for novel transcribed regions, 3,677 alternatively spliced exons, 3,353 3′-tiling probes, and 3,723 control probes. After data processing and normalization, boxplots of the raw and vsn normalized probe intensity values revealed no differences in samples (Fig. S1). None of the endometrial samples collected on day 14 of the estrous cycle clustered based on fertility classification, as all samples were relatively homogenous with respect to gene expression (Fig. 3). A heatmap based on pairwise correlations of the microarray data set is provided in Fig. 3A, and principal component analysis (PCA) is provided in Fig. 3B.

**Figure 4:**
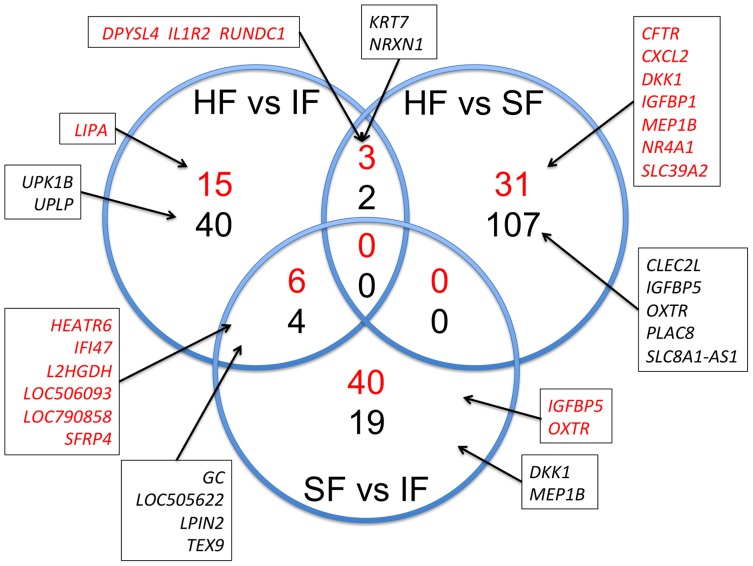
Venn diagram showing the number of unique or common transcripts between the endometrium of fertility-classified heifers (HF, high fertile; SF, subfertile; IF, infertile). Up-regulated (red) and down-regulated (black) genes are presented (P<0.01 and no false discovery rate with greater than 1.5-fold change). A few up- and down-regulated genes are highlighted in the boxes.

**Figure 5:**
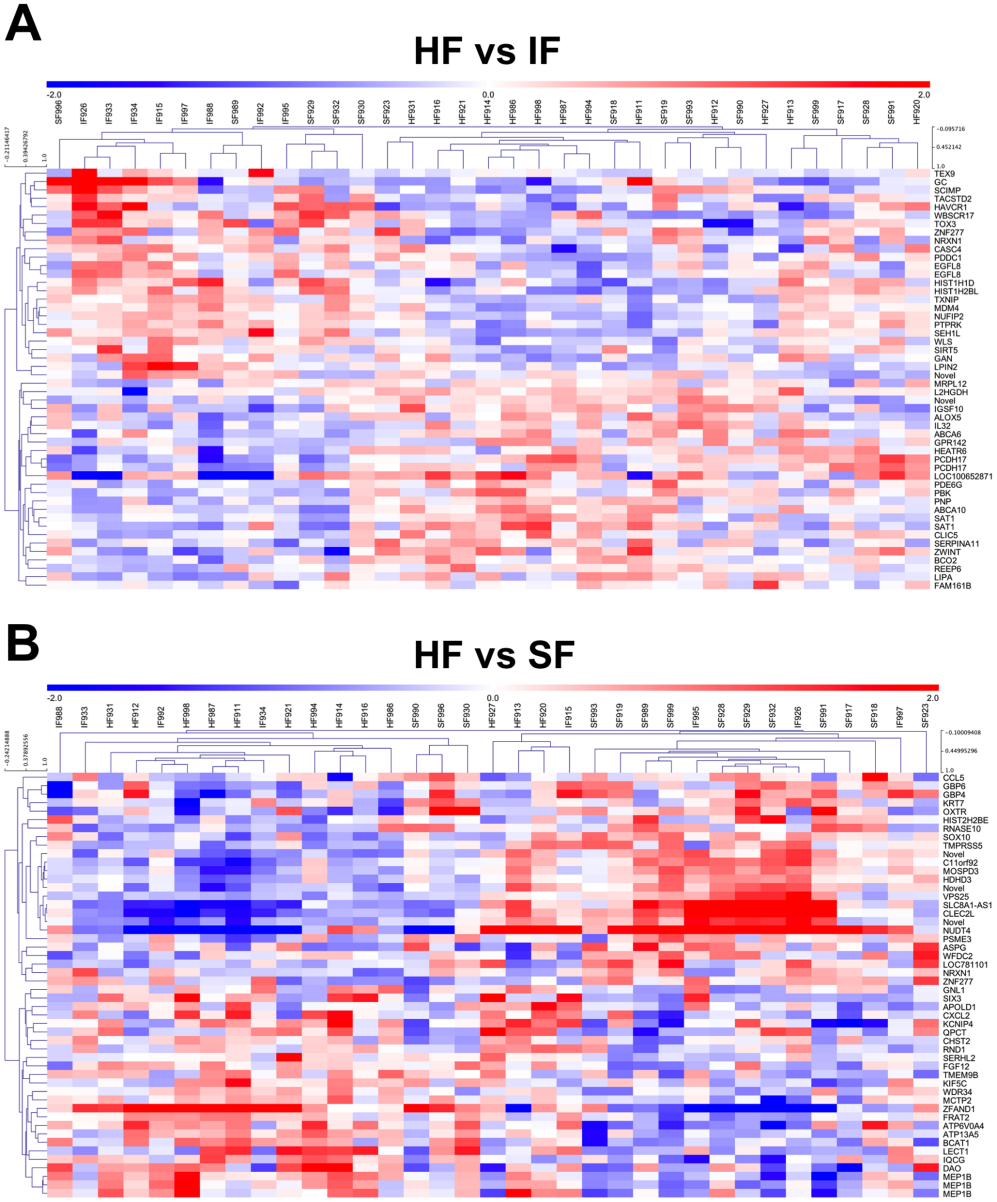
Hierarchical clustering analysis of differentially expressed genes in the endometrium of fertility-classified heifers. Up-regulated (red) and down-regulated (blue) genes are presented.

Statistical analysis of microarray data using BioConductor Limma revealed no probes with significant differences in signal intensity due to fertility classification. The lack of differences in the endometrium between fertility-classified heifers could be due to the use of algorithms designed for fewer replicates and homogenous differences between experimental groups [47]. Therefore, the data were reanalyzed with Bioconductor Limma with no false discovery rate (FDR). This analysis revealed many probes with nominal differences (*P*<0.01) in signal intensity based on fertility classification; the identity and description of up- and down-regulated probes is provided in Table S1. Real-time semi-quantitative PCR analysis of endometrial total RNA validated many nominally differentially expressed probes (Table 2). Venn diagrams of genes identified in the microarrays as being up- and down-regulated (>1.5-fold difference, nominal P<0.01) genes in the endometrium of different fertility group comparisons (HF vs IF, HF vs SF, SF vs IF) are presented in Fig. 4. Note the lack of overlapping differentially expressed genes in the comparisons of endometrium from HF vs IF and HF vs SF or HF vs SF and SF vs IF heifers; however, common up- and down-regulated genes were found in comparisons of the HF vs IF and SF vs IF heifers (Fig. 4). Hierarchical clustering was performed to identify similarly expressed genes in the different fertility-classified heifers using Pearson correlation coefficient analysis (Fig. 5 and Table S2).

**Table 2:** Comparison of endometrial mRNA levels for selected genes in the endometrium determined by microarray analysis and real-time semi-quantitative PCR (qPCR)^a^.

	HF vs IF		HF vs SF		SF vs IF	
Gene Symbol	Micro array	qPCR	Micro array	qPCR	Micro array	qPCR
*ASIP*	1.50	1.52	2.65^c^	3.03^c^	−1.77	−2.00
*GC*	−3.02^b^	−3.58^b^	−1.14	−1.36	−2.64^b^	−2.64^b^
*IGFBP1*	1.61	1.22	2.03^d^	2.20^d^	−1.26	−1.80
*IFI47*	2.71^c^	2.06^c^	−1.11	−1.08	3.01^c^	5.68^c^
*LECT1*	1.52^d^	1.21	1.82^d^	1.78^d^	−1.19	−1.44
*MEP1B*	1.40^d^	1.45	2.13^c^	1.88^c^	−1.52	−1.29
*NUDT4*	−2.64	−1.96^c^	−5.36^c^	−3.03^c^	1.02^d^	1.17
*OXTR*	1.02	1.22	−2.44^c^	−2.59^c^	2.47^c^	2.58^c^
*PLAC8*	−1.40	1.12	−1.93^d^	1.09	1.38	1.02
*RUNDC1*	5.19^c^	6.59^c^	5.88^c^	5.74^c^	−1.13	1.15
*SFRP4*	2.39^c^	2.30^c^	−1.11	−1.04	2.66^c^	2.39^c^
*RNF14*	−1.10	1.08	1.10	1.08	−1.21^d^	1.00
*XIST*	1.08	1.08	1.13	1.01	−1.04	1.07

a Data are presented as fold change.

b,c,d P-value for comparison (^b^ *P*<0.01, ^c^ *P*<0.05, ^d^ *P*<0.10).

Pathway analysis using DAVID revealed several overrepresented functional categories for differentially expressed genes in the endometria of HF as compared to IF (Table 3 and Table S4) and HF as compared to SF heifers (Table 4 and Table S5).

**Table 3:** Selected overrepresented functional categories for differentially expressed genes in High Fertile as compared to Infertile heifers (nominal P-value <0.01).

Representative enriched functional terms^1^	Enrich-ment Score^2^	No. Genes
***Selected terms for genes with lower expression in HF as compared to IF group***		
Macromolecular complex subunit organization (12, 2.8); chromatin (7, 5.7); ubl conjugation (9, 2.5); heterogeneous nuclear ribonucleoprotein M (6,2.6)	1.94	26
RNA binding (10, 2.1)	1.58	10
Topological domain:Lumenal (8, 3.0); signal-anchor (6, 2.4)	1.36	8
Zinc finger, RING-type (6, 3.2); Zinc finger, C3HC4 RING-type (5, 3.4)	1.24	6
Mitochondrion (11, 1.6); organelle envelope (9, 2.3); mitochondrial inner membrane (5, 2.6)	1.21	14
Manganese ion binding (4, 3.9)	1.15	4
***Selected terms for genes with higher expression in HF as compared to IF group***		
ATPase activity (5, 2.7); ATPase, AAA+ type, core (4, 5.1)	1.29	5
Protein kinase cascade (6, 2.9); MAPKKK cascade (6, 5.8); regulation of cellular protein metabolic process (6, 2.3)	1.22	10
Oxidation reduction (8, 2.2); oxidoreductase (7, 2.4)	1.18	8
Cell projection (8, 2.0); neuron projection (5, 2.6)	1.05	8
Lytic vacuole (4, 3.4); lysosome (4, 3.4)	1.02	4

1 in brackets: number of genes and fold enrichment of the functional term; ^2^geometric mean (in -log10 scale) of member's p-values of the corresponding annotation cluster.

**Table 4:** Selected overrepresented functional categories for genes with differential expression in High Fertile as compared to Subfertile heifers (nominal P-value <0.01).

Representative enriched functional terms^1^	Enrich-ment Score^2^	No. Genes
***Selected terms for genes with lower expression in HF as compared to SF group***		
Ubl conjugation (16, 2.3); isopeptide bond (11, 2.9); cross-link:Glycyl lysine isopeptide (Lys-Gly) (interchain with G-Cter in ubiquitin) (7, 2.9)	2.01	18
Steroid hormone receptor signaling pathway (5, 6.3); androgen receptor signaling pathway (4, 8.1)	1.91	5
Positive regulation of developmental process (10, 2.6); positive regulation of cell differentiation (9, 2.9)	1.87	10
Regulation of cellular localization (10, 2.9); regulation of secretion (7, 2.5); regulation of amine transport (4, 8.9)	1.85	11
Female pregnancy (5, 3.3); placenta development (5, 6.1); decidualization (3, 16.9)	1.72	7
Cell adhesion (18, 1.9)	1.72	18
Response to organic substance (16, 1.6); response to hormone stimulus (13, 2.6); response to steroid hormone stimulus (8, 3.0)	1.71	16
Negative regulation of cell communication (9, 2.7); negative regulation of signal transduction (8, 2.6)	1.60	9
Zinc finger (5, 2.7); Zinc finger, nuclear hormone receptor-type (4, 6.9); steroid hormone receptor activity (4, 6.1)	1.49	5
Cell cycle (19, 1.8); mitotic cell cycle (10, 2.0)	1.30	19
***Selected terms for genes with higher expression in HF as compared to SF group***		
RNA binding (25, 2.3)	3.59	25
mRNA metabolic process (19, 3.1); RNA splicing (17, 3.6); spliceosome (9, 4.1)	3.52	22
Ribonucleoprotein complex (29, 3.4); ribosome (13, 3.6); ribosomal protein (10, 3.5); translation (12, 2.2)	3.31	32
Organelle lumen (47, 1.6); nuclear lumen (36, 1.5); nucleoplasm (23, 1.6); nucleolus (22, 1.9)	2.17	48
Mitochondrion (23, 1.3); mitochondrial matrix (10, 2.7); mitochondrial ribosome (5, 6.3)	1.77	24
Methylation (9, 2.4); RNA recognition motif, RNP-1 (9, 2.7); Nucleotide-binding, alpha-beta plait (7, 2.1)	1.73	15
Endosome (11, 2.1); endocytosis (7, 2.2)	1.59	11
Microtubule binding (5, 4.5); tubulin binding (5, 3.3)	1.40	5

1 in brackets: number of genes and fold enrichment of the functional term; ^2^geometric mean (in -log10 scale) of member's p-values of the corresponding annotation cluster.

### Genome-wide Association Study (GWAS)

**Figure 6:**
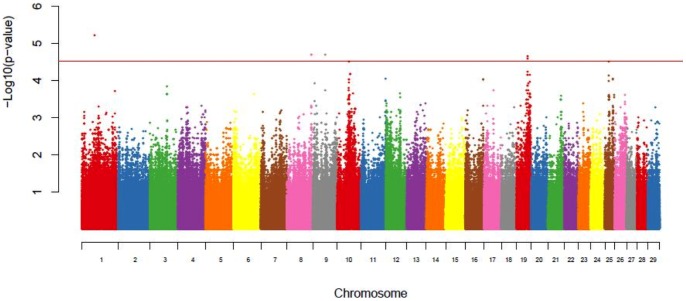
Genome-wide association or Manhattan plot of significance values for high fertile compared to low fertile heifers. The results of the genome-wide association analysis are shown for chromosomes 1 through 29 and the X chromosome (labeled as 30). The results are plotted by the −log_10_ significance values on the y-axis and the chromosomal location for each SNP tested on the x-axis. The red line represents the Wellcome Trust threshold for moderate evidence for significance association.

A GWAS study of DNA from 39 fertility-classified heifers (HF, n = 14; SF, n = 14; IF, n = 11) was conducted using the Illumina BovineHD BeadChip genotyping array. One heifer was removed from the GWAS due to a single nucleotide polymorphism (SNP) call rate of <95%, and two were removed as outliers following PCA, leaving 36 genotyped heifers. The SNPs were discarded if their minor allele frequency was <1% (27,819) or more than 10% of their genotypes were not called (47,559) or they failed the Hardy-Weinberg Equilibrium test (181 SNPs p<1×10^−10^), thereby leaving 707,971 SNPs for analysis. As summarized in Table 5 and illustrated in Fig. 6, moderate evidence for an association with fertility was found on BTA1 (p = 6.1×10^−6^), BTA8 (p = 1.99×10^−5^), BTA9 (p = 2.0×10^−5^), and BTA19 (p<2.7×10^−5^) by comparing high fertility (HF) and low fertility (SF and IF) heifers.

**Table 5:** SNPs associated with fertility.

Bovine Chromosome	Position (bp)	Significance(unadjusted)	Positional candidate gene(s)
BTA1	55,135,256	6.1×10^−6^	*LOC614129*
BTA8	106,991,900	1.99×10^−5^	*PAPPA*
BTA8	106,992,579	1.99×10^−5^	*PAPPA*
BTA9	47,513,052	1.99×10^−5^	*NDUFAF4, GPR63, FHL5, UFL*
BTA19	47,513,052	2.19×10^−5^	*EFCAB3, METL2, TLK2* *, *MRC2*
BTA19	47,559,874	2.19×10^−5^	*EFCAB3, METL2, TLK2* *, *MRC2*

* SNP associated with fertility resides within gene.

## Discussion

The present study found that four serial rounds of AI, each followed by pregnancy determination and termination on day 35, is an effective strategy to identify beef heifers with high and low rates of early pregnancy loss. Similarly, McMillan and Donnison [36] utilized 6 serial rounds of ET, each followed by pregnancy determination and then termination on day 35 of gestation, to identify dairy heifers with high and low fertility. That study suggested that failures in mechanisms involved in conceptus elongation and maternal recognition of pregnancy were the major cause of low fertility [36], [37]. Indeed, the majority of pregnancy losses in heifers, non-lactating cows and lactating cows occur during the period from fertilization to conceptus elongation [6], [24], particularly between days 7 and 16 of pregnancy [7]. Several studies in beef heifers and dairy cattle as well as sheep indicate that an early or delayed rise in circulating levels of progesterone after ovulation can advance or retard conceptus elongation [20], [26], [45]. In both the present study and that of McMillan and Donnison [36], minimal differences in ovarian follicular parameters or post-ovulatory circulating levels of progesterone were observed in the selected heifers, supporting the idea that differences in the maternal uterine environment between the HF and SF or IF heifers influence embryo survival. Results of the the present study in which heifers were embryo donors support the hypothesis that oocyte quality was not different among the fertility-classified heifers in the present study. Accordingly, the higher pregnancy rates to AI and as embryo recipients observed in the present study and that of McMillan and Donnison [36] for the HF heifers can be attributed to innate superior endometrial receptivity resulting in a uterus that was more competent to support growth and development of the conceptus for establishment of pregnancy. Because two embryos were transferred into each heifer in the present study, IFNT production should have been more than adequate to establish pregnancy if endometrial receptivity was sufficient.

In the present study, microarray analysis identified a number of differentially expressed transcripts in the endometria of fertility-classified heifers using samples collected on day 14 of the estrous cycle. The rationale for analyzing the endometria on day 14 post-estrus was that: (a) the majority of embryo loss in cattle occurs between days 7 and 16 of pregnancy [7]; (b) major gene expression changes in the endometrium that support elongation of the conceptus occur by day 13 in both pregnant and non-pregnant heifers [48]; and (c) differences in the endometrial transcriptome are not observed until days 15 or 16 in pregnant and non-pregnant heifers [48], [49]. Gene expression data from the present study supports the idea that patterns of endometrial gene expression are different in the endometrium among the fertility-classified heifers. Of note, differences in the endometrial transcriptome were observed between all different groups of heifers. The lack of conserved differences in the endometrial transcriptome of the HF versus low fertility (SF and IF) heifers indicates that the biological mechanisms underlying subfertility and infertility may be different. Of note, the functions of many of the nominally differentially expressed genes identified by microarray analysis of heifer endometria have not been investigated in the endometrium of ruminants or other mammals. For instance, both *RUNDC1* and *IFI47* mRNAs were more abundant in the endometria of HF as compared to IF heifers, and *RUNDC1* was also more abundant in endometria of HF than SF heifers. RUNDC1 (RUN domain containing 1) is a novel inhibitor of p53 and may have oncogenic activity [50], but has not been investigated in the uterus. IFI47 (Interferon gamma inducible protein 47) is induced by IFN gamma, but has not been investigated in the uterus; it has a purported biological role in control of protozoan parasitic infection. Analyses of the nominal differentially expressed genes using DAVID bioinformatics resources identified a number of functional categories that were different in the endometrial of fertility-classified heifers. Of note, genes associated with chromatin assembly and RNA binding were lower in HF than IF heifers (or higher in IF than HF heifers). In contrast, genes associated with cell signaling and metabolism were higher in HF than IF heifers (or lower in IF than HF heifers). Further, genes associated with ubiquitination, hormone receptor signaling and activity, and cell cycle were lower in HF than SF heifers (or higher in SF than HF heifers). Finally, genes associated with RNA binding, splicing and translation as well as mitochondrial function were higher in HF than SF heifers (or lower in SF than HF heifers). These pathways represent future areas of investigation for study of endometrial functions regulating conceptus elongation and associated with endometrial receptivity and successful pregnancy.

The temporal changes that occur in endometrial gene expression during the estrous cycle and early pregnancy of cattle have been recently published [33], [51]–[55]. In both cyclic and pregnant heifers, similar changes occur in expression of genes in the endometrium between days 7 and 13 post-estrus/mating, supporting the idea that the uterus develops a receptive endometrium to prepare for an expected pregnancy [56]. It is only in association with maternal recognition of pregnancy, which occurs around day 16 in cattle, that significant changes in the endometrial transcriptome are detectable between cyclic and pregnant animals, because the endometrium responds to increasing amounts of IFNT and likely prostaglandins secreted by the elongating conceptus [56]. Elevating levels of progesterone immediately after ovulation advances the normal changes in gene expression within the endometrium and stimulates conceptus elongation [51], [57]. Conversely, induction of low serum progesterone concentrations in heifers after ovulation [58] delayed the normal temporal changes that occur in the expression of genes in the endometrium [52], resulting in pregnancy loss. Ultimately, progesterone-induced changes in endometrial gene expression alter the composition of histotroph and, in turn, growth and development of the conceptus [57], [59]–[61]. The significance of endometrial secretions in acquisition of endometrial receptivity and embryo implantation is well documented in humans and domestic animals [28], [62], [63]. However, it is not well understood which genes and biological pathways in the endometrium are crucial to establish endometrial receptivity and support conceptus elongation in cattle. One approach to identify differentially expressed genes in the endometrium that contribute to the fertility phenotype is to determine which of them are up- or down-regulated in the endometrium of cattle between days 7 and 13 post-estrus/mating using data from previous studies [48], [51], [52]. Of the transcripts more abundant in HF than SF or HF than IF heifers, *DKK1, IGFBP1, and MEP1B* are increased between days 7 and 13 post-estrus/mating and regulated by progesterone in bovine endometria [64].

Insulin-like growth factor binding protein 1 (IGFBP1) was increased in the endometria of HF as compared to SF heifers. Further, IGFBP5 was more abundant in endometria of SF than IF heifers, but lower in HF than SF heifer endometria. IGFBPs prolong the half-life of the IGFs and have been shown to either inhibit or stimulate the growth promoting effects of the IGFs on cell culture. In sheep, IGFBP5 is expressed in the endometrial LE and GE of the ovine uterus [65]. In sheep and cattle, IGFBP1 is expressed specifically in the endometrial LE of the ovine and bovine uterus, a marker of endometrial receptivity and potential regulator of conceptus elongation [66], [67]. The only IGFBP with a RGD-integrin binding sequence is IGFBP1 [68]. The biological functions of IGFBP1 include stimulation of trophoblast cell migration [67], [69], [70] and inhibition of trophoblast invasiveness [71]. Integrins are expressed constitutively on both the conceptus trophectoderm and endometrial LE in sheep and cattle [72], [73] and are essential for blastocyst implantation but require functional binding and cross-linking and activation to regulate implantation [74], [75]. Both integrins and IGFBP1 are implicated in regulation of endometrial receptivity and implantation in humans [68], [76].

Meprin A, beta (MEP1B) was more abundant in the endometria of HF than SF heifers and was lower in the endometria of SF than IF heifers. MEP1B is a zinc metalloendopeptidase that is expressed by and secreted from epithelial cells, particularly the intestine and kidney (reviewed by [77]). In the bovine uterus, it is induced by progesterone in the endometrial glands and implicated in elongation of the conceptus [64]. Substrates for enzymatic cleavage by MEP1B include secreted phosphoprotein 1 (SPP1), gastrin releasing peptide (GRP), nidogen, fibronectin, neuropeptide Y and secretin, some of which are known components of ULF during early pregnancy in sheep [78], [79]. Cleavage of MEP1B substrates can lead to their degradation, however cleavage of other proteins leads to active forms such as cleavage of the pro-form of IL1B into active IL1B [77]. An additional role of meprins involves the cleavage of extracellular matrix proteins, which may be involved in adhesion of the conceptus trophectoderm to the endometrial epithelium.

Several of the differentially regulated transcripts in the fertility-classified heifers encoded factors involved in WNT signaling. Wingless-type MMTV integration site family member (WNT) genes are homologous to the Drosophila segment polarity gene wingless (wg). In humans and mice, the *WNT* family of genes encodes a group of 19 highly conserved secreted signaling molecules that are critical regulators of cell fate, growth, and differentiation, as well as cell-cell interactions [80]. Secreted FZD-related proteins (SFRPs) are forms of FZDs that contain the cysteine-rich domain but no transmembrane or intracellular segments and can bind WNTs to inhibit their activity [81], [82]. The four *DKK* (Dickkopf) genes encode secreted proteins that bind the FZD coreceptors and thus are antagonists of the WNT signaling pathway [83]. *SFRP4* mRNA was more abundant in HF than IF and SF than IF endometria, whereas *DKK1* mRNA was more abundant in HF than SF endometria but less abundant in SF than IF endometria. Of particular note, Cerri and coworkers [84] recently reported that DKK1 expression was increased in the endometria of day 17 pregnant as compared to nonpregnant cows and, interestingly, decreased in the endometria of lactating as compared to non-lactating cows on day 17 of pregnancy. The canonical and noncanonical WNT signaling pathways are thought to regulate conceptus elongation and growth in sheep [85]. The WNT signaling pathway is also crucial to implantation in mice [86]–[88], and failures in WNT signaling are associated with infertility in humans [89]. In human endometrium, DKK1 is up regulated in the endometrial stroma during the implantation window by progesterone [90], [91], and DKK1 secreted from decidual cells also plays a role in trophoblast cell invasion and outgrowth [92]. Indeed, activation of the canonical WNT signaling in bovine embryos retards their development in vitro, and DKK1 treatment can reduce those inhibitor effects [93]. Little is known about the expression or control of SFRP4 in the bovine uterus. SFRP4 expression is regulated by estrogen and progesterone and may act as a regulator of adult uterine morphology and function in rodents [94], [95]. Of note, women with repeated implantation failure after IVF treatment have lower levels of SFRP4 expression in their secretory phase endometria [96]. Further, DKK1 is aberrantly increased in endometria of women with excessive ovarian stimulation that may negatively affect implantation [97].

In the present study, *OXTR* mRNA was higher in the endometria of SF as compared to HF or IF heifers, but not different between HF and IF heifers. Expression of the OXTR increases substantially between days 13 and 16 in the endometrium of non-pregnant heifers [98], which is required for the endometrium to produce luteolytic pulses of PGF2α in response to pituitary- or luteal-derived oxytocin. Interestingly, Peterson and Lee [37] as well as Ledgard and coworkers [99] reported that cows could be selected for early pregnancy outcome by measuring uterine production of luteolytic PGF, with the superior cows producing less PGF2αin response to an oxytocin challenge on Day 16 of the estrous cycle. However, no differences in OXTR expression or genes involved in PG synthesis was observed in the superior and inferior pregnancy outcome cows selected by uterine PGF production [99]. Further, the predictive value of PG release in response to an oxytocin challenge was found not to be an effective indicator of subsequent pregnancy rates in cattle. Nonetheless, the increased OXTR expression in the endometria of SF heifers in the present study might be indicative of an asynchronous endometrial receptivity, which is detrimental to conceptus development [100]–[102].

Few genes were more abundant in the endometria of IF heifers. GC (group-specific component (vitamin D binding protein) was less abundant in the endometria of HF than IF heifers and also SF than IF heifers. The protein encoded by this gene belongs to the albumin gene family and binds to vitamin D and its plasma metabolites and transports them to target tissues [103]. GC has many physiologically important functions, ranging from transporting vitamin D3 metabolites, binding and sequestering globular actin and binding fatty acids to functioning in the immune system. Interestingly, Vitamin D is implicated in endometrial function and pregnancy success in humans and several animal models [104]. Collectively, the transcriptional profiling study highlights the complexity of gene expression in the endometrium and differences in endometrial function among the fertility-classified heifers. Future experiments will need to explore differences in the endometrial secrotome as it has a major influence on growth and development of histotroph growth and development of the conceptus [57], [59]–[61]. The significance of endometrial secretions in acquisition of endometrial receptivity and embryo implantation is well documented in humans and domestic animals [28], [62], [63].

The results of the GWAS and endometrial transcriptional profiling studies support the idea that fertility is a complex trait, which is reflected by the observed heterogeneity in gene expression patterns within groups of fertility-classified heifers [17]. The GWAS study here identified a number of SNPs with fertility associations and near or within candidate genes (Table 3). PAPPA (pregnancy-associated plasma protein A) is a secreted metalloproteinase that cleaves IGFBP4 and IGFBP5. Recent evidence indicates that PAPPA has an important role in modulating ovarian function and female fertility by control of the bioavailability of ovarian IGF [105]. Little is known about the other candidate genes identified in the GWAS with the exception of MRC2 (mannose receptor, C type 2) that has no viability or reproductive defects in homozygous null mice. Future validation studies are needed to determine if these SNPs can be used to select for fertility in heifer and cow populations. Improvement of functional traits using conventional approaches of quantitative genetics is difficult, because most reproductive traits are complex (polygenic) with low heritability [34], [35]. However, few GWAS or SNP studies of female fertility traits have been reported in cattle. One recent study explored relationships between production and fertility traits in dairy cattle via association studies of SNPs within candidate genes derived by expression profiling [106]. That study identified four SNPs with favorable effects on fertility and on yield traits, one SNP with favorable effects on fertility and percentage traits, and one SNP with antagonistic effects on two fertility traits. However, the genes used in the study were not represented in the candidate genes identified in the present study using a comprehensive SNP genotyping approach. Another study looked at genome-wide associations for fertility traits in Holstein-Friesan dairy cows using data from experimental research herds [107]. However, none of the SNPs identified in that study, which did evaluate number of services and pregnancy rate to first service, were the same as identified in the present study of fertility-classified beef heifers. The lack of similar SNP identification could be due to differences in breed, given that Holsteins are a highly selected breed, as well as reproductive history, disease history, and production parameters that would influence metabolism. Thus, future GWAS studies of female fertility need to be conducted with several different cattle breeds and utilize heifers or cows with defined parity and reproductive history as well as large population numbers.

## Conclusion

One of the major impediments to research on the genetics and biology of pregnancy in cattle is the lack of studies on animals with defined pregnancy loss. The present study and that of McMillan and Donnison [36] support the hypothesis that natural variation in pregnancy rates can be utilized in cattle to identify animals with innate differences in uterine competency support growth and development of the conceptus for establishment of pregnancy. The GWAS and transcriptional profiling studies are a first step towards understanding the genetic and biochemical determinants of endometrial receptivity and uterine function. Studies of animals with natural variation in uterine competency for pregnancy could help define which genes and biological pathways in the endometrium are crucial to establish endometrial receptivity and support conceptus elongation in cattle. Further, the use of this animal model could discover genes and biomarkers that can be used to select animals for higher fertility and to diagnose and treat subfertility and infertility.

## Supporting Information

Figure S1 **Boxplot of raw and vsn normalized probe intensity values.**
(TIFF)

Table S1 Analysis of the microarray data with Bioconductor Limma.
(XLS)

Table S2 Lists of transcripts clustered in the endometrium.
(XLS)

Table S3 qRT-PCR primers of candidate genes and housekeeping genes.
(DOC)

Table S4 Selected overrepresented functional categories for differentially expressed genes in High Fertile as compared to Infertile heifers (nominal P-value <0.01).
(XLS)

Table S5 Selected overrepresented functional categories for differentially expressed genes in High Fertile as compared to Subfertile heifers (nominal P-value <0.01).
(XLS)
