# Supplementary figures and images for: Effects of Fertility on Gene Expression and Function of the Bovine Endometrium
Source: PLoS One. 2013 Aug 5;8(8):e69444. doi: 10.1371/journal.pone.0069444 (PMC3734181; doi:10.1371/journal.pone.0069444)

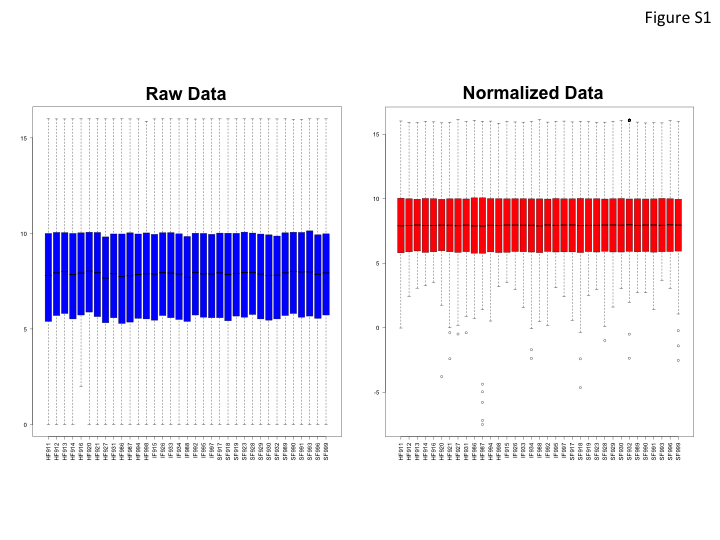

Supplement: Figure S1 — Boxplot of raw and vsn normalized probe intensity values. (TIFF) [file pone.0069444.s001.tiff]
